# A Dual-Stage Attention Model for Tool Wear Prediction in Dry Milling Operation

**DOI:** 10.3390/e24121733

**Published:** 2022-11-28

**Authors:** Yongrui Qin, Jiangfeng Li, Chenxi Zhang, Qinpei Zhao, Xiaofeng Ma

**Affiliations:** 1 Faculty of Engineering, The University of Sydney, Sydney, NSW 2006, Australia; 2 School of Software Engineering, Tongji University, Shanghai 201804, China; 3 College of Electronics and Information Engineering, Tongji University, Shanghai 201804, China

**Keywords:** tool wear prediction, attention mechanism, signal analysis, machine learning, gated recurrent unit

## Abstract

The intelligent monitoring of tool wear status and wear prediction are important factors affecting the intelligent development of the modern machinery industry. Many scholars have used deep learning methods to achieve certain results in tool wear prediction. However, due to the instability and variability of the signal data, some neural network models may have gradient decay between layers. Most methods mainly focus on feature selection of the input data but ignore the influence degree of different features to tool wear. In order to solve these problems, this paper proposes a dual-stage attention model for tool wear prediction. A CNN-BiGRU-attention network model is designed, which introduces the self-attention to extract deep features and embody more important features. The IndyLSTM is used to construct a stable network to solve the gradient decay problem between layers. Moreover, the attention mechanism is added to the network to obtain the important information of output sequence, which can improve the accuracy of the prediction. Experimental study is carried out for tool wear prediction in a dry milling operation to demonstrate the viability of this method. Through the experimental comparison and analysis with regression prediction evaluation indexes, it proves the proposed method can effectively characterize the degree of tool wear, reduce the prediction errors, and achieve good prediction results.

## 1. Introduction

With the continuous improvement and optimization of sensor technology, internet of things technology, and deep learning algorithms, the development of industrial intelligent manufacturing systems is more rapid, and constantly moving toward the integration of various emerging technologies. In the industrial intelligent manufacturing environment, most of the machining process is the cutting process, which inevitably causes tool wear. Tool wear refers to a process in which the metal material on the tool surface is continuously disappearing and the surface morphology is continuously changing due to the mechanical, chemical, and thermal effects of the cutting process [1].

The tool wear has an important impact on the machining process. When the tool is worn to the scrap state, but the machining process has still not stopped, it will damage the workpiece and even break down the machine tool, which may directly affect the processing efficiency, product quality, and production cost [2,3]. The traditional coping mode is to change tools at regular intervals, which can cause the waste of materials. In that case, the coping mode has gradually updated to intelligent tool changing based on the prediction of tool wear. Changing the severely worn tools through online monitoring and real-time prediction can not only improve tool utilization rate and processing quality, but also reduce safety accidents and shutdown rate.

In recent years, many scholars have conducted a lot of work on tool monitoring. Most of them are committed to the online monitoring of tool wear status and the prediction of remaining tool life usefulness. According to different measurement methods, the automatic monitoring solutions in tool wear can be mainly divided into two types, direct method and indirect method. The mainstream method is to use different sensors indirectly to collect digital signals, such as vibration, force, current, and acoustic emission. These signals can reflect the changes with time about the process of tool wear and can realize online intelligent monitoring without interrupting the machining process to establish a correlation between signal data and wear status, which is able to obtain the wear degree of the tool. Obviously, extracting useful features from original signals is critical for tool wear monitoring, and it can directly affect the prediction results of the model. Commonly used feature processing methods are time domain analysis, frequency domain analysis, and time–frequency domain analysis [4].

With the wide use of deep learning and neural network in various fields, the current research on tool wear mainly focuses on the combination of feature engineering and deep learning. Kong et al. [5] optimized the time–frequency statistic features and developed an artificial neural network model to predict the degree of tool wear. Tao et al. [6] presented a novel method based on the long short-term memory network (LSTM) and hidden Markov model to track the flank wear and predict the remaining useful life. Wu et al. [7] proposed a tool wear prediction model based on singular value decomposition (SVD) and bidirectional long short-term memory neural network (Bi-LSTM). SVD was used to extract the signal features from the reconstructed signal matrix. An et al. [8] used sparse auto-encoder and Pearson correlation coefficient to extract the sensitive features of the original cutting force signal and used these features to train back propagation neural network.

Although these studies have made great progress to improve the accuracy and reliability of the tool wear prediction model, there are still some limitations. In the feature extraction process of different sensors, only a few common features are extracted from experts’ domain knowledge, which can not be applied to more scenes or adjust different tools. In the process of feature selection, the influence degree between different features and target wear value is not reflected or ignored. The design of many neural network structures may cause the gradient attenuation between layers. Due to the instability and variability of the collected signal, the designed tool wear prediction model is not stable and accurate enough.

Hence, to solve these problems, and to accurately reflect the change in tool wear status with time during machining process, a dual-stage attention model is proposed to predict tool wear online. When the set threshold is reached, the machine will be stopped intelligently and change new tools. The main contributions of this paper include three parts:

Firstly, to obtain comprehensive signal characteristics, features are extracted from the collected sensor signals during milling cutter processing through three-domain analysis. Selecting highly relevant features by using the maximum information coefficient can reduce the redundant features and improve modelling efficiency.

Secondly, to extract deep features and reflect influence degree of different features, a network based on convolutional neural network (CNN) and bidirectional gated recurrent unit (BiGRU) with attention (CBGA) is proposed to encode feature vectors by applying the self-attention to assign different weights.

Finally, to improve the stability of tool wear prediction model, an IndyLSTM-attention network is proposed to predict the wear values.

The rest of the paper is organized as follows. Section 2 introduces related works about the application of deep learning in the tool wear prediction. A dual-stage attention prediction model is specifically described in Section 3. Section 4 evaluates the performance of the proposed model by a case study for tool wear prediction in a dry milling operation. Section 5 is the conclusion section of this paper.

## 2. Related Works

With the development of deep learning in the last few years, many deep learning methods based on predictive analysis have been widely adapted for the process of tool condition monitoring and tool wear prediction [9,10,11]. Dai, Zhang, and Meng used a stacked sparse auto-encoder network to reduce the feature vectors and build a least squares support vector machine prediction model based on cuckoo optimization parameters [12]. An adaptive method was developed by Cao, Sun, and Zhang by using a deep network to replace manual feature extraction from signals and proposed an on-line tool wear monitoring model based on convolution neural networks [13]. Wu, Jennings, and Terpenny introduced a method based on random forests for tool wear prediction [14]. To realize a real-time and accurate monitoring of the tool wear in machining process, Kong, Dong, and Chen presented a model based on the integrated radial basis function with kernel principal component analysis (KPCA_IRBF) and relevance vector machine (RVM) [15].

Considering the characteristics of the time series and dynamic changes of input data, the recurrent neural network introduces a cyclic structure, which can model dynamic time series data better than other neural networks. Therefore, RNN and its variations, such as LSTM and GRU, have been widely applied to this field of tool wear. For example, a recurrent neural network based on health indicator (RNN-HI) for RUL prediction of bearings was proposed by Guo, Li, and Jia [16]. Zhu, Xie, and Li established a tool wear monitoring model on the basis of long-term and short-term memory neural networks [17]. A deep neural network structure named convolutional bi-directional long short-term memory (CBLSTM) has been designed to address raw sensory data [18]. It presented a hybrid prediction scheme to solve long-term prediction problems by a newly developed deep heterogeneous GRU model, along with local feature extraction [19]. Inspired by the success of deep learning methods that redefine representation learning from raw data, Zhao, Wang, and Yan [20] proposed a network named local feature-based gated recurrent unit (LFGRU). It was a hybrid approach that combined handcrafted feature design with automatic feature learning for machine health monitoring.

Both GRU and LSTM are special RNN structures, which are proposed to solve the problems of gradient disappearance in RNN. Although these structures solve the gradient problems to some extent by using the tanh and sigmoid function as activation function, they will also cause gradient attenuation between layers. IndyLSTMs (independently recurrent long short-term memory cells) [21] were proposed on the basis of IndRNN [22], which adds the gate structure of LSTM. Compared with the traditional LSTM, the cyclic weight is no longer a full matrix, but a diagonal matrix. In each layer of IndyLSTM, the number of parameters and nodes shows a linear relationship, while the traditional LSTM is quadratic. This feature makes the model smaller and faster, and the accuracy of this model is better than the LSTM model in most cases. Therefore, the IndyLSTM is introduced to build a new network to solve the problems of gradient attenuation between layers and to obtain a stable and accurate model for tool wear monitoring.

Moreover, most tool wear prediction models based on the recurrent neural network mainly focus on the selection of input features, ignoring the influence degree of input features on the tool wear. The attention mechanism is widely used in various types of deep learning tasks, such as natural language processing, image recognition, and speech recognition. As a resource allocation mechanism, it can assign different weights to input features so that different features containing important information will not disappear with the increase in time steps, which can highlight the impact of more important information. In this way, full use of the network can help to study and improve the prediction quality for a longer period of stability [23].

In summary, existing works have used deep networks instead of traditional methods, such as manual or machine learning methods, to extract features from signals to improve the prediction accuracy. However, existing models, such as IndyLSTM, ignore the difference in the degree of influence of selected input features on tool wear. In addition, existing methods do not make full use of prior knowledge to improve model performance.

## 3. Dual-Stage Attention Prediction Model

**Figure 1:**
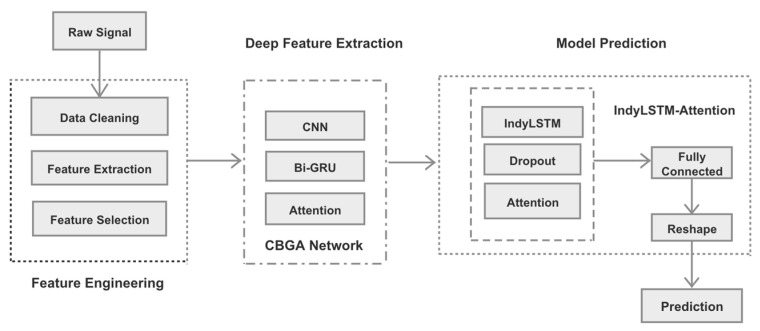
A dual-stage attention model for tool wear prediction.

The framework of the tool wear prediction model based on dual-stage attention is shown in Figure 1. The whole model mainly includes three layers: the feature engineering layer, the deep feature extraction layer, and the model prediction layer. After the initial feature engineering process to the raw signals, the CBGA network will be used to extract deep features. Finally, applying the IndyLSTM-Attention model to train and output the wear values generates a stable model to realize real-time prediction of tool wear.

### 3.1. Feature Engineering

**Figure 2:**
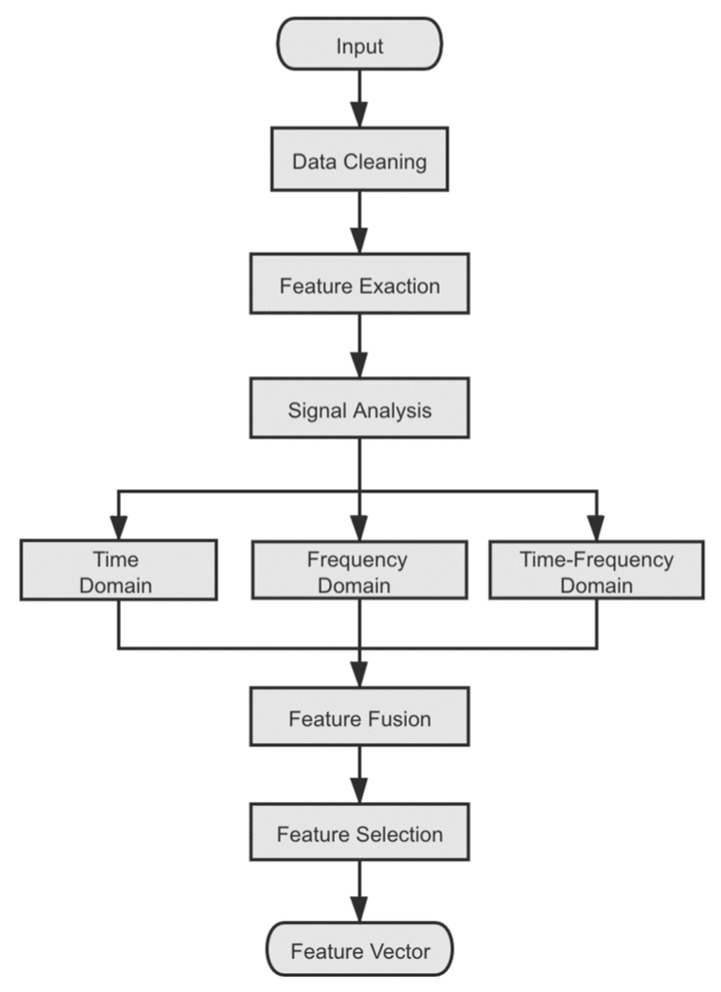
The flow of feature engineering.

The feature engineering layer consists of data cleaning, feature extraction, and feature selection. In this part, data cleaning mainly includes zero-averaging, removing the trend term, and normalizing signals. Meanwhile, according to the wavelet packet decomposition theory, high-frequency noise will be filtered out. Using the common signal analysis methods on the cleaned data, the statistical features of signal data are extracted from three domains. Combined with the existing research, this paper integrates time domain, frequency domain, and time-frequency to analyze the sensor signals comprehensively. After feature fusion, a preliminary feature selection is conducted on the extracted features. The flow of feature engineering is shown in Figure 2.

#### 3.1.1. Feature Exaction: Signal Analysis

Time domain analysis uses time axis as the coordinate to express the relationship between dynamic signals. It can effectively improve the signal-to-noise ratio and find the similarity and correlation of signal waveform transformations at different times. These obtained features can reflect the operating status of mechanical equipment.

Frequency domain analysis transforms the signals to the frequency axis. This method based on frequency characteristics makes up for the shortcomings of time domain analysis. It indirectly reveals the time domain performance of signals and easily displays the effect of system parameters on system performance. In this paper, spectrum analysis is used to analyze the signals after fast Fourier transform and to extract frequency domain features.

Wavelet analysis is a common time–frequency domain analysis method, which takes the signal information in both time domain and frequency domain into account. By analyzing frequency spectrum of sampled signal, the level of wavelet decomposition about signal is determined. The energy of each frequency band and the total energy entropy are taken as the time–frequency features after decomposition. In the Formula (1), $F_{s}$ is the sampling frequency, n is the number of layers, and $f_{min}$ is the minimum frequency band.

(1) $$f_{min}=F_{s}/2^{n+1}$$

The sampling frequency is 50 kHz, so a five-layer wavelet packet decomposition is performed. One then takes 2^5^ = 32 frequency band energy and energy entropy as time–frequency domain features. Thus, the signal analysis selects 13 features in the time domain, 3 features in the frequency domain, and 33 features in the time-frequency domain. In that case, 49 different features of each sensor channel will be extracted. The main extracted features are shown in Table 1.

#### 3.1.2. Feature Selection: Based on MIC

Unnecessary features will reduce training speed and generalization performance of the test set. In this paper, the features are selected and reduced by the maximum information coefficient (MIC). MIC can express various linear and non-linear relationships, and its value range is between 0 and 1. The higher the value, the stronger the correlation, so it has been widely used to select features in machine learning [24]. The basic principle of MIC utilizes the concept of mutual information, which is used to measure the degree of interdependence between two random variables. The mutual information can be explained as the following equation.

(2) $$I\left(x;y\right)=\int \;p\left(x,y\right)log_{2}^{\frac{p\left(x,y\right)}{p\left(x\right)p\left(y\right)}}dxdy$$

where p (x, y) is the joint probability density function of *x* and *y*, *p* (*x*) is the marginal probability density function of *x*, and *p* (*y*) is the marginal probability density function of *y*. The calculation formula of MIC is shown in Formula (3):

(3) $$MIC\left(x;y\right)=\begin{array}{c} max\\ a*b<B \end{array}\;\frac{I\left(x;y\right)}{log_{2}^{min\left(a,b\right)}}$$

In the above formula, *a* and *b* are the number of dividing grids in the *x* and *y* directions, *B* is a variable, and the size of *B* is generally set to 0.5 or 0.6 power of the total amount of data. Calculate the maximum information coefficient of tool statistical features and choose the target number of features according to the correlation and, at last, return the feature vectors after feature selection.

### 3.2. Deep Feature Extraction: CBGA Network

In this section, a CNN-BiGRU-attention (CBGA) network is proposed to encode and mine the deep features. It consists of CNN, Bi-GRU, and the attention mechanism, expanding the features in two dimensions of space and time.

CNN is a neural network with a deep structure that includes convolution calculations. It uses local connection and weight sharing to perform a higher-level and more abstract process on original data and can effectively extract local features of the data. CNN is mostly used for static output and is difficult for obtaining dynamic characteristics, especially when the data fluctuate or are unstable.

Bi-GRU can capture long-term dependencies and describe the continuous state output in time. It is suitable for analyzing time series data because of its memory function. The bidirectional structure can make full use of historical information and learn the dynamic laws of both positive and negative directions at the same time.

**Figure 3:**
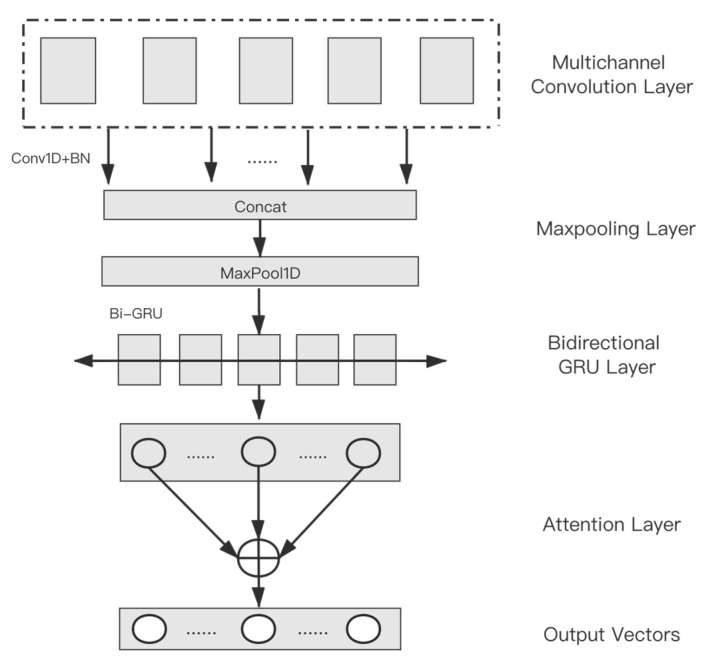
The structure of the CBGA.

Simultaneously, self-attention as one of the attention mechanisms can discover the internal characteristics of sequence data and highlight the important features. Thus, the self-attention layer is added to obtain final deep features. Based on the above theory, the CBGA network is designed to further process the feature vectors. Figure 3 shows the whole structure of the CBGA. As shown in this figure, the CBGA network can be divided into four parts: the multi-channel convolution layer, the max-pooling layer, the bidirectional GRU layer, and the attention layer.

Assume that the number of initially extracted features is *n*. The input data of this module is a n-dimensional feature vector that uses $I\left[i_{0},i_{1},\ldots i_{n}\right]$ to represent this input vector. Firstly, multi-channel convolution will be performed, it will connect the sequence results of the output, and it will obtain a *t*-dimensional vector C$\left[c_{0},c_{1},\ldots c_{t}\right]$, where *t* = k*f, *k* is the number of convolution layers, and *f* is the number of filters of the convolution neural network.

The max-pooling operation will be carried out. After that, the full sequence feature extraction will be conducted on the input through the bidirectional GRU. This part output G$\left[g_{0},g_{1},\ldots g_{m}\right]$ is the concatenation of the results of forward GRU and backward GRU, where *m* = 2*h, and *h* is the number of Bi-GRU’s hidden units. At last, the attention value of each GRU node will be calculated in the attention layer. A weighted feature vector Z$\left[z_{0},z_{1},\ldots z_{m}\right]$ is obtained as the final deep feature encoding vector.

Using mathematical formulas to express the self-attention mechanism, the input sequence from the Bi-GRU layer is G$\left[g_{0},g_{1},\ldots g_{m}\right]$, and the output sequence is Z$\left[z_{0},z_{1},\ldots z_{m}\right]$. Obtain three sets of vector sequences through linear transformation:

(4) $$Q=W_{Q}G\in {\mathbb{R}}^{d_{3}\times N}$$

(5) $$K=W_{K}G\in {\mathbb{R}}^{d_{3}\times N}$$

(6) $$V=W_{V}G\in {\mathbb{R}}^{d_{2}\times N}$$

where *Q*, *K*, and *V* are query vector sequence, key vector sequence, and value vector sequence, respectively. $W_{Q}$,$W_{K}$, and $W_{V}$ are the parameter matrix that can be learned, respectively. In the definition of self-attention, *Q* = *K* = *V* = *G*, so the output vector $z_{i}$ is calculated as:

(7) $$z_{i}=att((K,V),q_{i})=\sum_{j=1}^{N}\alpha_{ij}v_{j}=\sum_{j=1}^{N}softmax(s(k_{j},q_{i}))$$

where *i*, *j* ∈ [1, *m*] are the positions of the output and input vector sequences, and $s\left(k_{j},q_{i}\right)$ is a function to calculate the similarity between two vectors.

### 3.3. Model Prediction: IndyLSTM-Attention

The IndyLSTM-attention model is used in this paper to train and output the prediction. The model consists of the IndyLSTM network, the attention network, and the fully connected network, and these decode the feature sequences and output the required prediction. In the common RNN decoding unit, generally only the last sequence is taken from the output result as the prediction result. However, other sequences in the network structure are also meaningful. Combining other sequences through the attention mechanism may help us achieve a better fitting effect.

Therefore, the Bahdanau attention [25] is added after the IndyLSTM layer. The formula of Bahdanau attention mechanism is as follows:

(8) $$att((K,V),q)=\sum_{i=1}^{N}\alpha_{i}v_{i}=\sum_{i=1}^{N}\frac{exp(s(k_{i},q))}{\sum_{j}exp(s(k_{j},q))}v_{i}$$

**Figure 4:**
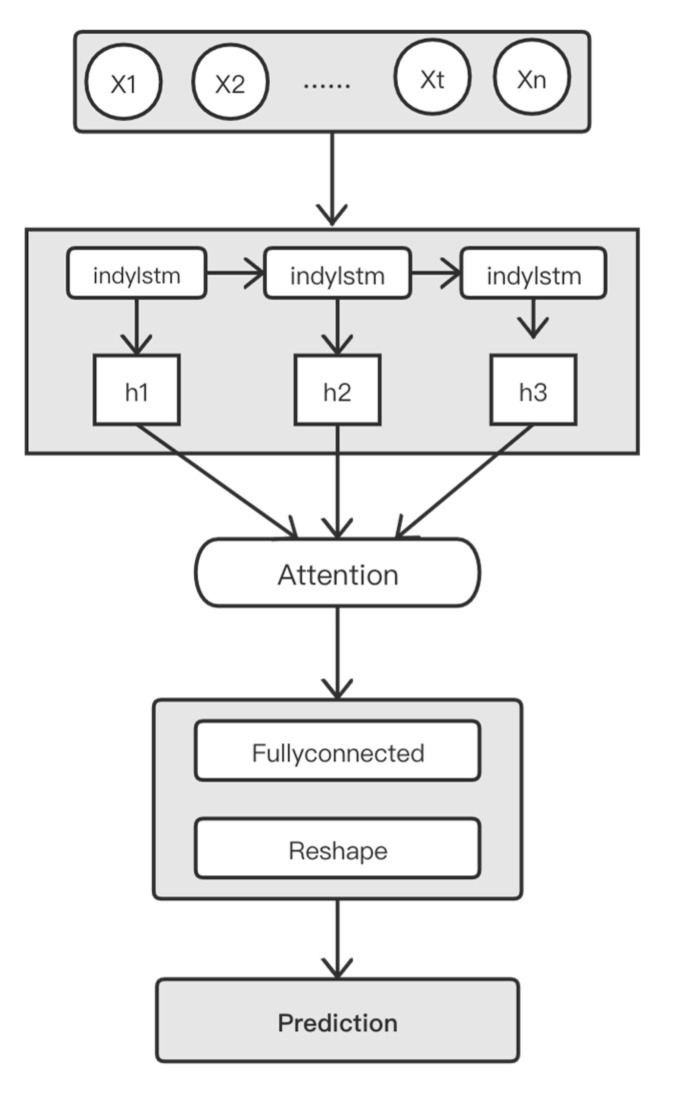
The architecture of the IndyLSTM-Attention model.

Input the deep feature results *Z* [$z_{0},z_{1},\ldots z_{m}$] extracted by the CBGA network into the model. The IndyLSTM-Attention network will output the vector *H*$\left[h_{0},h_{1},\ldots h_{u}\right]$, where *u* is equal to the number of hidden units of the IndyLSTM cell. At the fully connected network, two layers of fully connected networks are used to get the prediction. The architecture of the IndyLSTM-Attention model is shown in Figure 4.

## 4. Experiments

In this section, an empirical evaluation is conducted to test the performance of the proposed model. The descriptions of the datasets and experimental setup are introduced in detail. The proposed model is compared with other common prediction methods to form the comparison results and discussion.

### 4.1. Descriptions of Datasets

Open datasets were used to verify the predictive performance of the model, which were collected from the ball end carbide milling cutter of a high-speed (CNC) machine operated under dry milling operations [26]. Each training record contains one “wear” file that lists the flank wear values measured for three cutting edges after each cut in 10^−3^ mm and a folder with approximately 300 individual data acquisition files (one for each cut). The data acquisition files have seven columns of dynamometer, accelerator, and acoustic emission data. The main equipment and cutting parameters are specifically listed in Table 2.

In the experiment, six independent milling cutters (c1~c6) were used for the full tool life test. The force sensor, acceleration sensor, and acoustic emission sensor were used to collect signals, and each tool collected seven channel signals, which include force in three directions (*X*, *Y*, *Z*), vibration in three directions (*X*, *Y*, *Z*), and AE–RMS. During the experiment, all the sensor data were collected on a data acquisition card, and the data acquisition card transmitted all the information to the computer. Meanwhile, in the process of cutting the workpiece, the tool would be stopped in every cutting and used the microscope to measure the wear in the *X*, *Y*, and *Z* directions. The test was terminated when the tool was severely worn out and could not work anymore. 315 samples were obtained during the test, taking the average of the flank wear in three directions as the true value of tool wear estimation, and the unit of the tool wear is 10^−3^ mm.

In the given datasets, c1, c4, and c6 are training data with corresponding wear values, and c2, c3, and c5 are test data without wear values. Therefore, during the process of model verification, c1, c4, and c6 were selected as the training dataset. The leave-one-out method was adopted to achieve cross-validation by using two datasets as training set and using the rest one for verification. Therefore, three different test cases can be created, denoted as C1, C4, and C6. The partition of datasets is shown in Table 3.

### 4.2. Evaluation Index

In order to quantify the performance of all comparison methods, three commonly used evaluation indicators were selected to evaluate the regression loss, including mean absolute error (MAE), root mean square error (RMSE) and the coefficient of determination ($R^{2}$ score). Among the selected functions, both the MAE and $R^{2}$ scores are relatively robust and insensitive to outliers and noise. On the contrary, RMSE can integrate the advantages and disadvantages of MSE and MAE, is very sensitive to extremely large or small errors, and can make the model tend to be optimal.

MAE is the average of absolute values of errors. RMSE is the square root of mean of all squared errors. These two metrics are calculated as follows:

(9) $$MAE=\frac{1}{n}\sum_{i=1}^{n}\left|\bar{y_{i}}-y_{i}\right|$$

(10) $$RMSE=\sqrt{\frac{1}{n}\sum_{i=1}^{n}{\left(\bar{y_{i}}-y_{i}\right)}^{2}}$$

where $y_{i}$ and $\bar{y_{i}}$ are true and predicted tool wear values.

The $R^{2}$ score is the coefficient of determination, reflecting how much of the fluctuation of *y* can be described by the fluctuation of *x*. The value range of the $R^{2}$ score is between 0 and 1. The closer the value is to 1, the higher the degree of interpretation of the variable. The expression is as follows:

(11) $$R^{2}=1-\frac{\sum {\left(y_{i}-y_{i}\right)}^{2}}{\sum {\left(y_{i}-\bar{y}\right)}^{2}},where\;\bar{y}=\frac{1}{n}\sum_{i=1}^{n}y_{i}$$

### 4.3. Evaluation Setup

The vibration sensor signals were chosen for modeling. According to the part of feature exaction, the signal analysis was performed from three directions (*X*, *Y*, and *Z*) of vibration signals. Therefore, 147-dimensional (49*3) features were obtained in total. Then, the 40 best features were selected with high correlation by MIC, and the feature vectors were obtained after preprocessing. Meanwhile, the average of the wear value (mm) in three directions after each cutting was taken as true wear value of the tool. The initial values of the parameters of the experimental models are set with reference to the pre-trained models. In the experiment, the parameters are adjusted one by one by fixing other parameters and fine-tuning one parameter until the optimal result is obtained. The specific structure and experimental parameter configurations of the model are shown in Table 4.

The IndyLSTM-attention model was also compared with common neural networks including RNN, LSTM, GRU, IndRNN, and IndyLSTM. These different neural network methods are used as the model to output tool wear values after training. The input vectors of these models are extracted by CBGA network, and the size of hidden units in recurrent neural cells is unified to be the same as 128. The loss function used is mean squared error (MSE). Stochastic gradient descent (SGD) is adopted as an optimizer algorithm to train the models. During the training process, by using the estimator to build a deep recurrent network model, it can easily configure, train, and evaluate various machine learning models.

### 4.4. Experimental Evaluation

**Figure 5:**
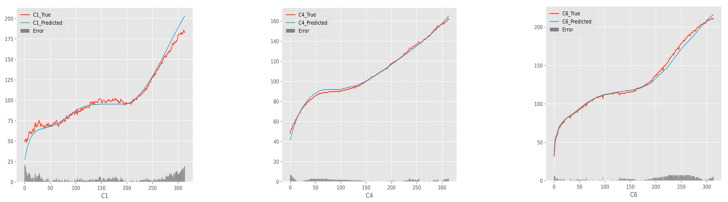
The tool wear prediction curve of a dual-stage attention model.

This section shows the results of comparison experiments and makes a brief analysis. This gives the prediction curves of the proposed method for three different test sets, as shown in Figure 5. In the figure, the broken line is the actual value of tool wear, the smooth curve is the predicted value of tool wear, and the bottom histogram is the error between the predicted value and the true value.

Table 5 shows all the results of common methods on three test cases, including the RMSE, MAE and $R^{2}\;\mathrm{score}$.

**Figure 6:**
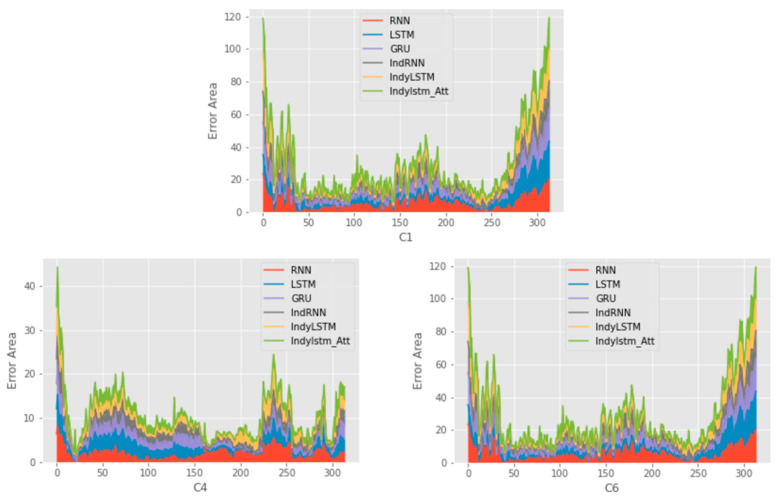
The area chart of error absolute value of six models on three cases.

Figure 6 shows the error area between the true wear values and the predicted values. The area chart can clearly display the error size of each prediction model.

**Figure 7:**
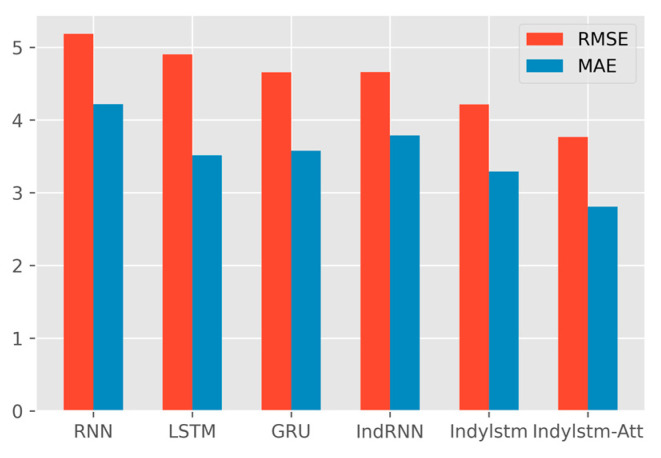
The area chart of error absolute value of six models for three cases.

In order to show the comparison results more intuitively, the average performance of six methods is calculated for three test cases, as shown in Table 6. The average performance comparison histogram of three cases is shown in Figure 7.

Overall, the model proposed by this paper has a good fitting effect, and the curves basically match the true data from Figure 5. The prediction of C4 and C6 is better than C1. According to the comparison results of Table 5 and Table 6, the IndyLSTM-attention in three indicators has the best performance. It can be observed that the IndyLSTM and IndyLSTM-attention outperforms the RNN, LSTM, and GRU neural network in three cases. This shows that independently recurrent long short-term memory networks perform better than traditional recurrent neural networks in this situation. Meanwhile, the comparison between the results of IndyLSTM and IndyLSTM-attention also shows that all indicators have been improved to a certain extent. Therefore, it can be concluded that the accuracy of prediction can be improved by adding an attention layer to the predictive model.

Further analysis is seen through the error area chart, the area where the prediction error of the proposed model is the smallest. At the same time, the model is found to have large fluctuations at the beginning and at the end of the tool prediction, and the error of other stages is small. Finally, through the histogram, the differences between different models can be observed. RMSE and MAE of the proposed model are much smaller than in the other models.

## 5. Conclusions

In this paper, an IndyLSTM model with a self-attention mechanism is proposed in order to solve the problem that existing deep learning methods ignore (the different influences of the degree of input features on tool wear in the process of intelligent tool wear monitoring). By using the 2010 PHM Society Conference Data Challenge open datasets, the proposed model has achieved better performance than common regression prediction methods in all three evaluation criteria (MAE, RMSE, and $R^{2}$ score). Through experimental verification, there are two main findings obtained.

(1) By applying the self-attention mechanism in the deep feature extraction and tool wear prediction model to assign different weights to different input features, performance of the prediction model for tool wear can be effectively improved.
(2) By combining prior experience, the feature selection method using the maximum information coefficient can effectively reduce redundant features, which shows an ability to improve modeling efficiency.

However, the features used in this paper are a combination of time domain, frequency domain, and deep learning features, wherein the time and frequency domain features are dependent on prior knowledge. The future work is to select better time and frequency domain features and better feature selection criteria to further improve the performance of the model.

## Figures and Tables

**Table 1:** The information of extracted features.

Domain	Feature Name	Formula	Feature Name	Formula
Time	mean	$\bar{y}=1/n\sum_{i=1}^{n}y_{i}$	form factor	$\frac{\sqrt{\left(1/n\sum_{i=1}^{n}y_{i}{}^{2}\right)}}{1/n\sum_{i=1}^{n}|y_{i}|}$
	root mean square	${\left(\frac{1}{n}\sum_{i=1}^{n}y_{i}{}^{2}\right)}^{1/2}$	spectral kurtosis	$\frac{E\left[{\left\{\left(y-\bar{y}\right)/\sigma \right\}}^{4}\right]}{{\left(\sqrt{1/n\sum_{i=1}^{n}y_{i}{}^{2}}\right)}^{4}}$
	maximum	Max$\left\{\left|y_{i}\right|\right\}$	spectral skewness	$\frac{E\left[{\left\{\left(y-\bar{y}\right)/\sigma \right\}}^{3}\right]}{{\left(\sqrt{1/n\sum_{i=1}^{n}y_{i}{}^{2}}\right)}^{4}}$
	variance	$\frac{1}{n}\sum_{i=1}^{n}{\left(y_{i}-\bar{y}\right)}^{2}$	impulse index	$Max\left\{\left|y_{i}\right|\right\}/\bar{y}$
	skewness	$E\left[{\left\{\left(y-\bar{y}\right)/\sigma \right\}}^{3}\right]$	clearance index	$\frac{Max\left\{\left|y_{i}\right|\right\}}{{\left(1/n\sum_{i=1}^{n}|y_{i}|\right)}^{2}}$
	kurtosis	$E\left[{\left\{\left(y-\bar{y}\right)/\sigma \right\}}^{4}\right]$	standard deviation	[$\frac{1}{n}\overset{n}{\underset{i=1}{\sum }}{\left(y_{i}-\bar{y}\right)}^{2}{]}^{1/2}$
	crest factor		$\frac{max\left(y\right)-min\left(y\right)}{\sqrt{1/n\sum_{i=1}^{n}{\left(y_{i}\right)}^{2}}}$	
Frequency	centroid frequency	$FC=\frac{\sum_{f=1}^{n/2}fw\left(f\right)}{\sum_{f=1}^{n/2}w\left(f\right)}$	frequency variance	$\frac{\sum_{f=1}^{n/2}{\left(f-FC\right)}^{2}w\left(f\right)}{\sum_{f=1}^{n/2}w\left(f\right)}$
	mean square frequency		$MSF=\frac{\sum_{f=1}^{n/2}f^{2}w\left(f\right)}{\sum_{f=1}^{n/2}w\left(f\right)}$	
Time-Frequency	wavelet energy	$E_{i=1,2,\ldots 32}=wt_{\emptyset }^{2}\left(i\right)/n$	wavelet entropy	E = $\sum_{i=1}^{32}wt_{\emptyset }^{2}\left(i\right)/n$

**Table 2:** The main equipment and cutting parameters of milling experiment.

Machine Tool	Roders Tech RFM760	Spindle Speed	10,400 r/min
Cutter	Ball end Carbide milling	Feed rate	1555 mm/min
Milling material	Stainless steel HRC52	Cutting width	0.125 mm
Dynamometer	Kistler 9265B	Cutting depth	0.2 mm
Vibration sensor	Kistler 8636C	Sampling frequency	50 kHZ
Collector	NI DAQ PCI 1200	Charge amplifier	Kistler 5019A
Wear gauge	Microscope LEICA MZ12	Cooling condition	Dry Machining

**Table 3:** The setup of training and testing data.

Group	Training Set	Testing Set
C1	c4, c6	c1
C4	c1, c6	c4
C6	c1, c4	c6

**Table 4:** Model structure and parameter configuration of this paper.

Structure	Layer
CBGA	Conv1D (filters = 128, kernel_size = 2) Batch Normalization
	Conv1D (filters = 128, kernel_size = 3) Batch Normalization Conv1D (filters = 128, kernel_size = 4) Batch Normalization
	MaxPool1D (pool_size = 5, strides = 1)
	Bi-GRU (hidden_units = 64)
	Self-Attention
Model	IndyLSTM (hidden_units = 128) Dropout (0.8) Attention (attention_length = 22) Fully Connected Reshape

**Table 5:** The MAE, RMSE and R^2^ for all methods for three test cases.

Models	C1			C4			C6		
	RMSE	MAE	$R^{2}$	RMSE	MAE	$R^{2}$	RMSE	MAE	$R^{2}$
RNN	7.111	5.611	0.955	2.360	1.983	0.993	6.080	5.060	0.979
LSTM	6.535	4.391	0.959	2.414	2.024	0.992	5.757	4.130	0.981
GRU	6.378	4.855	0.963	2.020	1.731	0.994	5.568	4.150	0.983
IndRNN	6.216	5.129	0.964	2.037	1.702	0.995	5.728	4.537	0.982
IndyLSTM	6.180	4.499	0.965	1.977	1.632	0.995	4.485	3.745	0.988
IndyLSTM-Att	**5.796**	**4.242**	**0.970**	**1.822**	**1.406**	**0.996**	**3.681**	**2.776**	**0.992**

The bold face indicates the best performance.

**Table 6:** The average performance of the three cases.

Models	RMSE	MAE	$R^{2}$
RNN	5.184	4.218	0.976
LSTM	4.902	3.515	0.977
GRU	4.655	3.579	0.980
IndRNN	4.660	3.789	0.980
IndyLSTM	4.214	3.292	0.983
IndyLSTM-Att	**3.766**	**2.808**	**0.986**

The bold face indicates the best performance.

## Data Availability

The dataset and parameter configuration used to support the findings of this study are included within the article.

## References

[1] Xu X., Wang J., Zhong B., Ming W., Chen M. (2021). Deep learning-based tool wear prediction and its application for machining process using multi-scale feature fusion and channel attention mechanism. Measurement.

[2] He Z., Shi T., Xuan J., Li T. (2021). Research on tool wear prediction based on temperature signals and deep learning. Wear.

[3] Ambhore N., Kamble D., Chinchanikar S., Wayal V. (2015). Tool Condition Monitoring System: A Review. Mater. Today Proc..

[4] Zeng H., Thoe T.B., Li X., Zhou J. Multi-modal Sensing for Machine Health Monitoring in High Speed Machining. Proceedings of the 2006 4th IEEE International Conference on Industrial Informatics 2006.

[5] Liu M.K., Tseng Y.H., Tran M.Q. (2019). Tool wear monitoring and prediction based on sound signal. Int. J. Adv. Manuf. Technol..

[6] Tao Z., An Q., Liu G., Chen M. (2019). A novel method for tool condition monitoring based on long short-term memory and hidden Markov model hybrid framework in high-speed milling Ti-6Al-4V. Int. J. Adv. Manuf. Technol..

[7] Wu X., Li J., Jin Y., Zheng S. (2020). Modeling and analysis of tool wear prediction based on SVD and BiLSTM. Int. J. Adv. Manuf. Technol..

[8] Shi C., Panoutsos G., Luo B., Liu H., Li B., Lin X. (2018). Using Multiple-Feature-Spaces-Based Deep Learning for Tool Condition Monitoring in Ultraprecision Manufacturing. IEEE Trans. Ind. Electron..

[9] Li X., Ding Q., Sun J.-Q. (2018). Remaining useful life estimation in prognostics using deep convolution neural networks. Reliab. Eng. Syst. Saf..

[10] Xiao P., Zhang C., Luo M. (2018). Modeling method for tool wear prediction based on ADNLSSVM. China Mech. Eng..

[11] Roy S.S. (2015). An Application of ANFIS-Based Intelligence Technique for Predicting Tool Wear in Milling. Intelligent Computing and Applications.

[12] Dai W., Zhang C., Meng L. (2020). Support vector machine milling wear prediction model based on deep learning and feature re-processing. Comput. Integr. Manuf. Syst..

[13] Cao D.L., Sun H.B., Zhang J.D., Mo R. (2020). In-process tool condition monitoring based on convolution neural network. Comput. Integr. Manuf. Syst..

[14] Wu D., Jennings C., Terpenny J., Gao R.X., Kumara S. (2017). A Comparative Study on Machine Learning Algorithms for Smart Manufacturing: Tool Wear Prediction Using Random Forests. J. Manuf. Sci. Eng..

[15] Kong D., Chen Y., Li N., Duan C., Lu L., Chen D. (2019). Relevance vector machine for tool wear prediction. Mech. Syst. Signal Process..

[16] Guo L., Li N., Jia F., Lei Y., Lin J. (2017). A recurrent neural network based health indicator for remaining useful life prediction of bearings. Neurocomputing.

[17] Zhu X., Xie F., Li N. (2019). Tool wear state monitoring based on long-term and short-term memory neural network. Manuf. Technol. Mach. Tools.

[18] Zhao R., Yan R., Wang J., Mao K. (2017). Learning to Monitor Machine Health with Convolutional Bi-Directional LSTM Networks. Sensors.

[19] Wang J., Yan J., Li C., Gao R.X., Zhao R. (2019). Deep heterogeneous GRU model for predictive analytics in smart manufacturing: Application to tool wear prediction. Comput. Ind..

[20] Zhao R., Wang D.Z., Yan R.Q., Mao K.Z., Shen F., Wang J.J. (2018). Machine Health Monitoring Using Local Feature-Based Gated Recurrent Unit Networks. IEEE Trans. Ind. Electron..

[21] Gonnet P., Deselaers T. Indylstms: Independently Recurrent LSTMS. Proceedings of the ICASSP 2020-2020 IEEE International Conference on Acoustics, Speech and Signal Processing (ICASSP) 2020.

[22] Li S., Li W., Cook C., Zhu C., Gao Y. Independently Recurrent Neural Network (IndRNN): Building A Longer and Deeper RNN. Proceedings of the IEEE Conference on Computer Vision and Pattern Recognition 2018.

[23] Peng W., Wang J., Yin S. (2019). Short term load forecasting model based on attention-lstm in power market. Grid Technol..

[24] Kinney J.B., Atwal G.S. (2014). Equitability, mutual information, and the maximal information coefficient. Proc. Natl. Acad. Sci. USA.

[25] Bahdanau D., Cho K., Bengio Y. (2014). Neural machine translation by jointly learning to align and translate. arXiv.

[26] PHM Society Conference Data Challenge. https://phmsociety.org/phm_competition/2010-phm-society-conference-data-challenge/.

